# Diversity and seasonality of horse flies (Diptera: Tabanidae) in Uruguay

**DOI:** 10.1038/s41598-019-57356-0

**Published:** 2020-01-15

**Authors:** Martín Lucas, Tiago K. Krolow, Franklin Riet-Correa, Antonio Thadeu M. Barros, Rodrigo F. Krüger, Anderson Saravia, Cecilia Miraballes

**Affiliations:** 1 Instituto Nacional de Investigación Agropecuaria (INIA), Plataforma de Salud Animal, Tacuarembó, Uruguay, Casilla de Correo 78086, Tacuarembó, CP 45000 Uruguay; 2 Facultad de Veterinaria, Universidad de la Republica (UDELAR), Alberto Lasplaces 1620, CP 11600 Montevideo, Uruguay; 3 Universidade Federal do Tocantins – UFT, Rua 03, Qd 17, S/N, Bairro Jardim dos Ipês, Porto Nacional, TO Brazil; 4 Embrapa Beef Cattle, Campo Grande, MS Brazil; 5 Universidade Federal de Pelotas, Instituto de Biologia, Departamento de Microbiologia e Parasitologia, Campus Universitário Capão do Leão, s/n°, Pelotas, RS Brazil

**Keywords:** Software, Ecological epidemiology

## Abstract

Horse flies (Diptera: Tabanidae) cause direct and indirect losses in livestock production and are important vectors of pathogens. The aim of this study was to determine the diversity and seasonality of horse fly species at an experimental farm in Tacuarembó and the diversity of species in different departments of Uruguay. For 20 months, systematic collections were performed in two different environments at the experimental farm using Nzi and Malaise traps. In addition, nonsystematic collections were performed at farms located in the departments of Paysandú, Tacuarembó and Colonia. A total of 3,666 horse flies were collected, and 16 species were identified. These species included three species that had not been previously recorded in Uruguay, namely, *Dasybasis ornatissima* (Brèthes), *Dasybasis missionum* (Macquart), and *Tabanus aff*. *platensis* Brèthes, and a species that had not been previously taxonomically described (*Tabanus* sp.1). Among the systematically captured samples, the most abundant species were *Tabanus campestris* Brèthes, *T*. *aff*. *platensis* and *D*. *missionum*, representing 77.6% of the collected specimens. The horse fly season in Tacuarembó started in September and ended in May. No horse flies were caught during winter. Variations in the prevalences of species in the different departments were observed, which indicates the need for new sampling efforts.

## Introduction

Horse flies (Tabanidae) are hematophagous dipterans that cause direct losses to livestock production due to irritation, stress, and blood loss in animals, particularly cattle and horses^1^. In fact, horse flies have directly resulted in decreases in the weight gain of cattle of 0.1 to 1 kg per day^1,2^. Economic losses are directly related to the number of horse flies present in the environment^3^. In addition to the losses caused by the direct effects of horse flies, these pests cause indirect losses due to their role as a mechanical vector of numerous pathogens, including those causing bovine leukosis, vesicular stomatitis, equine infectious anemia, swine fever, anthrax, and tularemia, as well as various species of trypanosomes and *Anaplasma marginale*^4,5^. These indirect losses might be even more important than the direct losses. Not all members of the Tabanidae family have the same potential for transmitting disease agents because they exhibit various hematophagous behaviors and present different anatomical characteristics, which determine the amount of blood that they can transport^5–7^.

According to the Köppen classification, Uruguay has a subtropical climate with four marked seasons, a mean annual temperature of 17.29 °C and a mean humidity of 76.03%^8^. Uruguay, along with the state of Rio Grande do Sul (Brazil) and the province of Buenos Aires (Argentina), comprise the Pampa biome.

The emergence of the first generation of horse flies depends on the latitude and the season^9,10^. Horse flies are active mostly in warm seasons when both the relative humidity and temperature are high. Only females are hematophagous, and the adult longevity varies from two to three weeks^1^. Although the females need a blood meal every three or four days, their bites are painful. Animals attempt to remove the flies, which might result in interruptions in feeding by the flies. This interruption causes female flies to seek other animals to complete their meal, and these flies are able to fly several kilometers and reach speeds of 5 m/s, which highlights their potential as a mechanical vector^11^.

A wide variety of traps have been used to study the diversity and abundance of horse fly species in different environments^12,13^. However, the Nzi trap was designed specifically to catch biting flies^14^, such as horse flies and stable flies (Stomoxyinae). Previous studies have shown that this trap can capture a greater number of some horse fly species than other traps^15^, whereas other genera of horse flies tend to be captured in higher numbers by other traps^1^.

Approximately 4300 species belonging to 137 genera of horse flies have been described worldwide^16,17^. The Tabanidae family is subdivided into four subfamilies, namely, Chrysopsinae, Pangoniinae, Scepsidinae and Tabaninae, and the subfamilies Chrysopsinae and Tabaninae are considered the most relevant to the mechanical transmission of pathogens^7,18^. In the neotropical region, 71 genera and 1205 species have been described^16,17,19^, and 350 and 480 species of Tabanidae have been identified in Argentina^20^ and Brazil, respectively^21^.

In Uruguay, 43 species belonging to 14 genera have been reported^17^; however, because most of these captures were performed in the XIX and XX centuries, whether this diversity of horse flies still exists in the country is unclear. Additionally, the abundance, ecology and seasonality of the Tabanidae family in Uruguay have not been previously investigated. Therefore, the objectives of this study were to evaluate the seasonality of the horse fly species present at an experimental farm in Tacuarembó and the diversity of species in different departments of Uruguay.

## Results

During the study, 3,666 horse flies were collected: 3,211 were obtained through systematic collections, and 455 were acquired via nonsystematic methods. These specimens represented two subfamilies, three tribes, six genera and 16 species. Fourteen species were definitively identified taxonomically, one species was preliminarily identified as *Tabanus affinity platensis* Brèthes (*T*. *aff*. *platensis*), and an undescribed species of *Tabanus* (*Tabanus* sp.1) was also found. In addition, 11 individuals belonging to the genus *Tabanus* could not be taxonomically identified, but these individuals are likely members of the same species and were thus classified as *Tabanus* spp.

### Systematic collections

Among the 3,211 specimens captured by this method, a total of 15 species were identified, and three of these species (*Tabanus campestris* Brèthes; *T*. *aff*. *platensis*; *Dasybasis missionum* (Macquart)) represented 77.6% of the total samples captured (Table 1). The other identified species with varying relative abundances were a previously undescribed species (*Tabanus* sp.1), *Tabanus triangulum* Wiedemann, *Tabanus acer* Brèthes and *Poeciloderas quadripunctatus* (Fabricius). The remaining species only accounted for 3.0% of the captures, and 3.4% of the individuals could not be identified due to their poor condition.

**Table 1:** Number of individuals and species of horse flies caught by traps using the systematic collection protocol between October 2017 and June 2019.

Species	Lowland			Highland			TOTAL (%)
	Traps						
	NZI 2	NZI 3	MAL 5	NZI 1	NZI 4	MAL 6	
	Number of individuals						
*Catachlorops circumfusus*	0	1	0	0	2	1	4 (0.1)
*Chrysops brevifascia*	1	7	2	1	0	0	11 (0.3)
*Dasybasis missionum*	96	564	21	2	14	18	715 (22.3)
*D*. *ornatissima*	0	1	0	0	0	0	1 (0.0)
*Poeciloderas lindneri*	0	17	0	0	0	1	18 (0.6)
*P*. *quadripunctatus*	3	30	0	0	0	0	33 (1.0)
*Tabanus acer*	9	31	1	6	12	3	62 (1.9)
*T*. *aff*. *platensis*	3	837	0	14	11	2	867 (27.0)
*T*. *campestris*	28	863	2	5	6	7	911 (28.4)
*T*. *claripennis*	1	17	0	0	5	0	23 (0.7)
*T*. *fuscofasciatus*	3	16	0	0	1	0	20 (0.6)
*T*. *fuscus*	1	17	1	0	0	0	19 (0.6)
*T*. *pungens*	0	0	0	0	0	1	1 (0.0)
*T*. sp.1	59	203	3	23	40	14	342 (10.7)
*T*. *triangulum*	5	24	3	27	14	3	76 (2.4)
Unidentified	6	87	9	1	3	2	108 (3.4)
TOTAL (%)	215 (6.7)	2715 (84.5)	42 (1.3)	79 (2.5)	108 (3.4)	52 (1.6)	3,211 (100)

Traps located in the “lowland” habitat were responsible for 92.5% of the total catches obtained during the study, and within this habitat, one of the traps (Nzi 3) captured 84.5% of the total specimens (Table 1). The most abundant species in the “lowland” habitat were *T*. *campestris* (30.0%), *T*. *aff*. *platensis* (28.3%) and *D*. *missionum* (22.9%). The individuals captured in the “highland” habitat represented only 7.5% of the total, and the three most abundant species were *Tabanus* sp.1 (32.2%), *T*. *triangulum* (18.4%) and *D*. *missionum* (14.2%).

**Figure 1:**
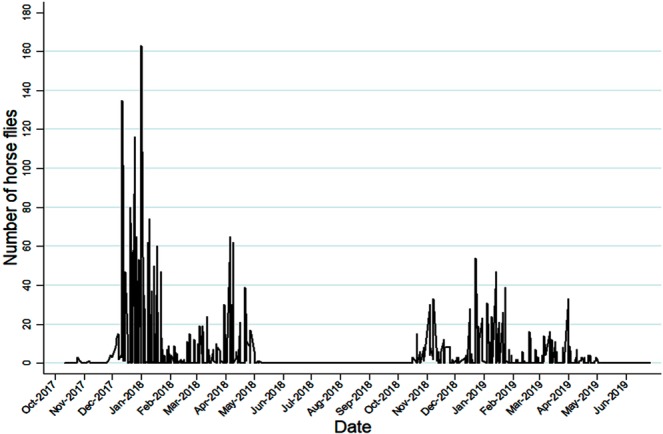
Number of horse flies caught in the “lowland” environment by systematic captures during the study period.

Figure 1 depicts the seasonality of the horse flies in the “lowland” habitat, where most individuals were caught, and shows that these flies were active from the end of September to the beginning of May and exhibited no activity from June to August. This figure also shows the presence of three peaks: one at the end of spring, another one in mid-summer (the most pronounced one), and a third one during the fall. A decrease in the total captures during the second season was also observed.

**Figure 2:**
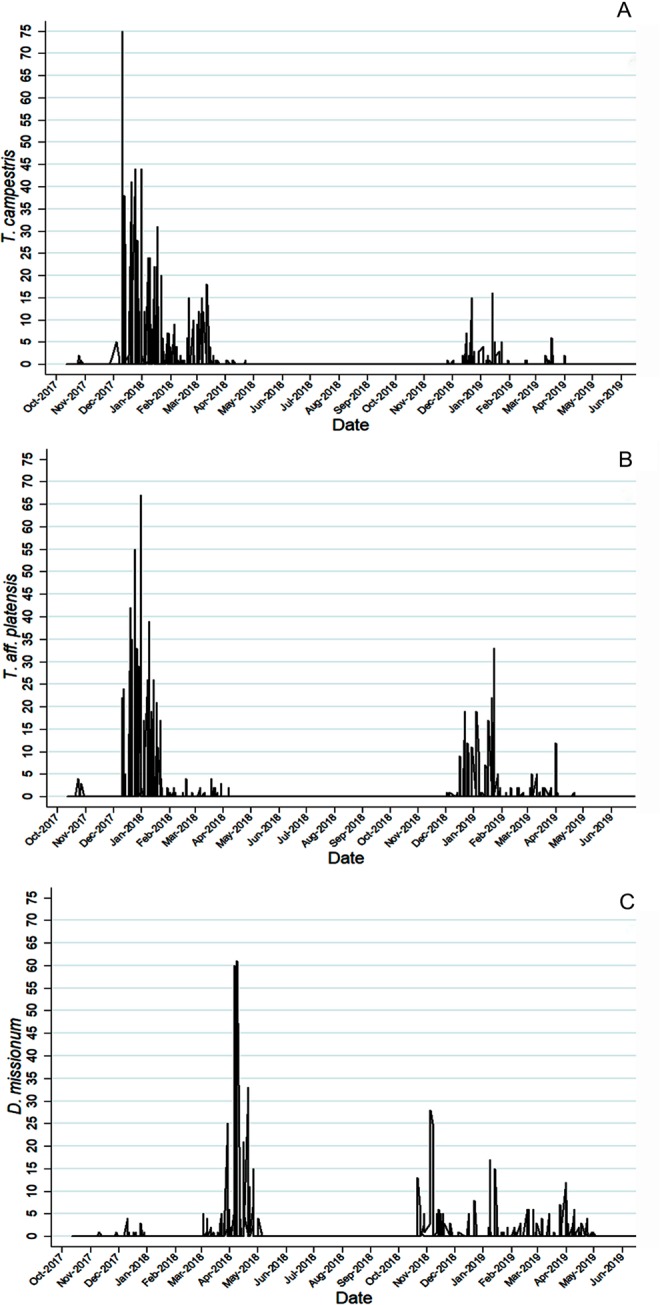
Seasonality of the three most frequently captured species using the systematic collection method: *T*. *campestris* (**A**), *T*. *aff*. *platensis* (**B**) and *D*. *missionum* (**C**).

Among the total captures in both habitats, 2,336 individuals were captured during the first season of horse fly activity (September 2017-May 2018), whereas 875 horse flies were captured during the second season (September 2018-May 2019). Figure 2 shows the different seasonality of the three most prevalent species. As indicated in the figure, *D*. *missionum* showed a different behavior, presenting peaks during late spring and autumn.

Nzi traps were responsible for 97.1% of the collections, whereas Malaise traps captured 2.9%. In the “lowlands”, the Malaise trap captured 1.4% of the total individuals, while in the “highlands”, it was responsible for 21.7% of the collections. Among the 15 species captured in the systematic collections, *Tabanus pungens* Wiedemann was the only species not captured by Nzi traps, whereas four of the 15 species, namely, *P*. *quadripunctatus*, *Tabanus fuscofasciatus* Macquart, *Tabanus claripennis* (Bigot) and *Dasybasis ornatissima* (Brèthes), were not captured by Malaise traps.

**Figure 3:**
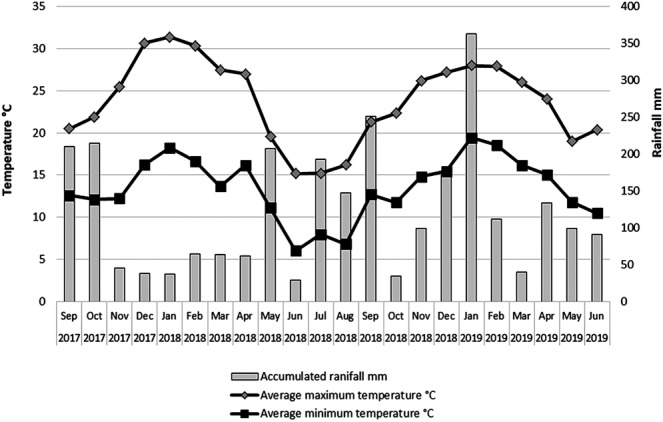
Monthly values of the average maximum temperature, average minimum temperature and accumulated rainfall throughout the study period.

Throughout the study period, the monthly average temperature ranged from 10.4 °C to 25.0 °C, the average minimum temperatures were between 6.0 °C and 19.4 °C, and the average maximum temperature ranged from 15.2 °C to 31.4 °C. The accumulated rainfall during this period was 2715.3 mm, and the average relative humidity varied between 56% and 90% (Fig. 3; Supplementary Table 1). During the first season (September 2017-May 2018), the accumulated rainfall was 944.8 mm, whereas that in the second season (September 2018-May 2019) was 1309.6 mm, indicating a clear difference in rainfall between the seasons, particularly during the capture peak in the summer (December-January).

**Figure 4:**
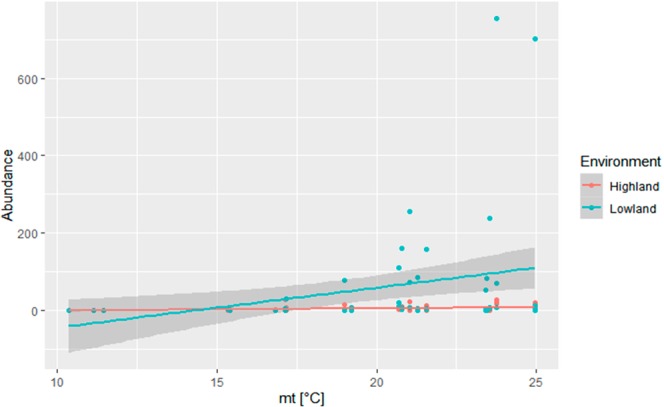
Abundance of horse flies in Tacuarembo, Uruguay, as a function of the mean temperature (°C) (mt), month, and year according to a GLM analysis with a quasi-Poisson distribution. The models for each environment are ${{\rm{Abundance}}}_{{\rm{Highland}}}={\exp }^{-6.886+0.386\ast {\rm{mt}}}$ and ${{\rm{Abundance}}}_{{\rm{Lowland}}}={\exp }^{-4.391+0.386\ast {\rm{mt}}}$.

An analysis of the response variable of the abundance of horse flies captured by the traps during the entire study period revealed that the average minimum and maximum temperatures, the relative humidity and the rainfall did not have a significant effect (p > 0.05), and these variables were thus removed from the final model. The significant differences obtained in the study were due to increases in the mean temperature (mt) and the environment. The mt (Chi_1;129_ = 3,989.3, p < 0.001) and environment (Chi_1;128_ = 2,675.1, p < 0.001) influenced the variations in the abundances of horse flies (Fig. 4), and no interaction was found between these variables. According to the abundance models, the abundance of horse flies in the lowland environment was 12-fold higher than that in the highland environment, and individuals were predicted to be present at average temperatures of 18 °C and 12 °C in the highland and lowland habitats, respectively. The models predicted a relative abundance of approximately 200 individuals per trap at a temperature of 25 °C in the lowland environment, whereas in the highland habitat, approximately 15 individuals were predicted to be found in each trap.

### Nonsystematic collections

**Figure 5:**
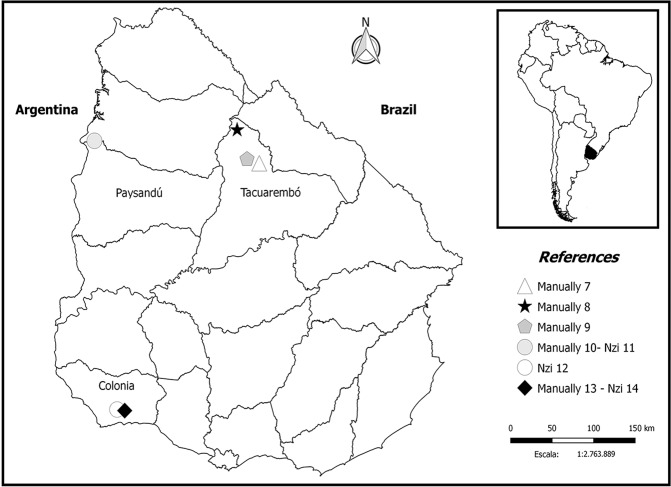
Location and capture method used for nonsystematic collections.

A total of 455 individuals were collected in the departments of Colonia (n = 343), Tacuarembó (n = 74) and Paysandú (n = 38) (Fig. 5; Table 2).

**Table 2:** Number of individuals and species collected using the nonsystematic collection protocol with traps or manually in the departments of Paysandú, Colonia and Tacuarembó.

Capture method	Tacuarembó			Paysandú		Colonia			Total
	Manually 7	Manually 8	Manually 9	Manually 10	NZI 11	NZI 12	Manually 13	NZI 14	
Species	Number of individuals (%)								
*Chrysops brevifascia*	0	0	1 (7,7)	0	0	0	0	0	1
*Dasybasis missionum*	1 (2,0)	10 (100,0)	0	0	0	0	0	15 (27,3)	26
*Dichelacera unifasciata*	0	0	5 (38,4)	0	0	0	0	0	5
*Poeciloderas lindneri*	0	0	0	3 (75,0)	13 (38, 2)	256 (90,2)	4 (100)	10 (18,2)	286
*P*. *quadripunctatus*	0	0	1 (7,7)	0	0	0	0	0	1
*Tabanus acer*	7 (13,7)	0	0	0	0	0	0	0	7
*T*. *aff*. *platensis*	0	0	2 (15,4)	0	2 (5,9)	0	0	1 (1,8)	5
*T*. *campestris*	2 (3,9)	0	0	0	1 (3,0)	0	0	0	3
*T*. *claripennis*	0	0	0	0	0	4 (1,4)	0	2 (3,6)	6
*T*. *fuscofasciatus*	0	0	0	0	18 (52,9)	0	0	0	18
*T*. sp.1	41(80,4)	0	4 (30,8)	1 (25,0)	0	0	0	0	46
*T*. spp.	0	0	0	0	0	6 (2,1)	0	5 (9,1)	11
*T*. *triangulum*	0	0	0	0	0	0	0	22 (40,0)	22
Unidentified	0	0	0	0	0	18 (6,3)	0	0	18
TOTAL	51	10	13	4	34	284	4	55	455

In Colonia, 78.7% of the captured species were *Poeciloderas lindneri* (Kröber), whereas the two most frequently captured species in Paysandú were *T*. *fuscofasciatus* (47.4%) and *P*. *lindneri* (42.1%). The analysis of the manually captured samples in the Tacuarembó department revealed that the most abundant species was the undescribed species *Tabanus* sp.1 (60.8% of the captures).

This sampling method resulted in the collection of a total of 12 species, and *Dichelacera unifasciata* Macquart was the only species not caught using the systematic collection protocol. Eleven individuals classified as *Tabanus* spp. were also captured, but their taxonomic identification was not achieved.

## Discussion

Of the 14 species that were taxonomically identified, *D*. *ornatissima* and *D*. *missionum* had not been previously observed in Uruguay, even though both have been recorded in Argentina and *D*. *missionum* has been previously observed in Brazil^10,16^. A final identification of *T*. *aff*. *platensis* via comparison with “type” specimens is necessary. *Tabanus platensis* has not been previously reported in Uruguay, but its presence is expected because its distribution in Argentina includes Chaco, Santa Fe, Entre Ríos and Buenos Aires^16^.

Two of the three new records for Uruguay (*D*. *missionum* and *T*. *aff*. *platensis*) were among the three most prevalent species found during the systematic collection and were not found to be abundant species in similar studies in southern Brazil^10^. This study also identified a previously undescribed species (*Tabanus* sp.1), which will be described elsewhere.

The findings that the captures were concentrated between September and May and that horse fly activity was not detected during winter differ from previous observations in southern Brazil, where horse flies were still obtained during winter, albeit at a markedly lower abundance^10^. Southern Brazil exhibits a peak in horse fly abundance during spring and a gradual decrease as winter approaches, but it is still possible to capture horse flies during this season^10^. In the Tacuarembó department, which belongs to the same Pampa biome as southern Brazil, the largest number of horse flies was observed during summer, and lower peaks were obtained during spring and autumn. This variation can be explained by climatological differences between zones or years as well as differences in the species of horse flies captured in the two regions^10,22,23^. In Tacuarembó, the peak recorded in autumn was mostly due to *D*. *missionum*. However, this peak was not obtained in Rio Grande do Sul, where *D*. *missionum* was not among the most abundant species^10^. It is important to note that different species respond differently to variations in climatic factors^15^.

Throughout the study period, only the mt and the environment exerted statistically significant effects on the number of captured individuals. In contrast, some studies have related the abundance of horse flies with an increase in relative humidity^24^ interacting with the mt^10^ and highlight that the influence of this variable differs among species^10,15^.

The environment was a determining factor related to the number of horse flies collected. On average, the traps located in “lowland” habitats, which is characterized by native forest and creeks, captured a 12-fold higher number of horse flies than the traps located in the “highland” habitats. These findings could allow a more efficient use of traps for mitigating horse fly attacks on commercial farms by through their use in environments with a greater number of horse flies. In addition, grazing management patterns that preclude the animals from grazing in the most infested paddocks during population peaks could be established to prevent the simultaneous presence of hosts and parasites^1,2^. One study conducted in the Pantanal biome (Brazil) found no difference between the captures performed in areas composed of native forests and those carried out in grasslands^25^. However, studies conducted in Santa Barbara, Pará (Brazil)^26^, found differences in the abundance and diversity of species captured in two environments, namely, in areas near a forest and in areas composed of clear fields (grassland). Among the specimens captured, 71.5% belonged to the environment near a forest, and 28.5% were found in clear fields. In addition, 23 of the 47 species were present in both environments, whereas 14 were exclusively found in near a forest, and nine were only found in grasslands.

Even when the first season of captures occurred during a dry summer and the second occurred during a rainy summer, a similar pattern in population peaks was observed. However, a marked decrease in the number of individuals caught was observed during the second season of systematic collection. This decrease in captures during the second season might have been influenced by the constant presence of the traps; however, no notable decrease in the density of horse fly populations in the environment was observed in previous studies that used traps as a control method^1,2,27^. Another possibility is that this decrease was related to climatological and/or environmental factors, such as the amount of rainfall during the second year, which could affect the abundance and activity of horse flies, as well as the trap efficiency.

The variations in the prevalences and the species captured in different regions denote the need to continue collecting horse flies in different areas of the country. For example, in the department of Colonia, the most prevalent species found was *P*. *lindneri*, whereas this species accounted for less than 1% of the total captures in the department of Tacuarembó obtained using the systematic collection protocol. Additionally, the importance of capturing new individuals in Uruguay, particularly in rural areas, is important for assessing whether the diversity of species reported was not changed by urbanization^17^.

Horse flies are detrimental to livestock production due to their direct effects on animals and their ability to act as vectors for at least 35 pathogens^1,4,5^. The relevant agents of disease that are potentially transmissible by horse flies in Uruguay include *A*. *marginale*, bovine leucosis virus, *Brucella abortus*, *Leptospira* spp., and equine infectious anemia virus. More studies are needed to determine the role of the Tabanidae species found in Uruguay on the epidemiology of these agents.

## Materials and Methods

During a 20-month period from October 2017 to June 2019, systematic collections were performed using four Nzi traps and two Malaise traps at the Experimental Farm of INIA La Magnolia (EFILM) in the department of Tacuarembó (31°42′29.0″S, 55°48′09.1″W). Two Nzi traps and one Malaise trap were placed in a “lowland” habitat with native forest and creeks, whereas the other two Nzi traps and one Malaise trap were placed in a “highland” habitat, which consisted of a clearer environment near a cultivated eucalyptus forest. The minimum distance between traps within the same environment was 250 m, whereas the maximum distance between them was 1170 m. Traps located in different environments were separated by at least 560 m and not more than 2350 m. All the traps remained active during the 20-month experimental period and maintained in the same relative positions with no rotation. The traps were used without baits of any kind. According to a conventional climatological station located at the EFILM, the following experimental parameters were recorded on a monthly basis: average maximum and minimum temperatures, mt, average mean relative humidity and accumulated rainfall.

In addition, nonsystematic collections were performed on farms located at the departments of Paysandú, Tacuarembó and Colonia using Nzi traps and/or manually with plastic jars. These captures were carried out to expand the diversity of horse fly species collected. A defined collection time pattern was not established, and the capture methods varied; thus, these data were not used in the analyses of the seasonality and abundance of the horse fly species.

Among the total number of horse flies captured, samples from one to 10 specimens of each suspected species were prepared for taxonomic identification.

### Data collection

#### Systematic collections

Between October and December 2017, two Nzi traps (Rincon-Vitova, USA) (Nzi 1 and Nzi 4) and one Malaise trap (Rincon-Vitova, USA) (Mal 6) were placed in a clear field environment close to an artificial eucalyptus forest (highland), and two Nzi traps (Nzi 2 and Nzi 3) and one Malaise trap (Mal 5) were placed in an environment close to creeks and native forest (lowland). Because alcohol was not used in the collectors for the conservation of horse flies, during summer (December to February), which is the season with the highest populations of horse flies, collections were performed at least three times a week to prevent desiccation of the captured specimens. During winter (June to August), collections were performed at least once a week, whereas in spring (September to November) and autumn (March to May), collections were carried out one to three times per week depending on the number of specimens collected.

#### Nonsystematic collections

To identify species in different areas of the country or species that might not be captured by traps, sporadic samplings were performed in the departments of Tacuarembó (three farms), Paysandú (one farm) and Colonia (two farms) (Table 3). These samplings were performed manually and/or with Nzi traps. Using the manual sampling protocol, some horse flies were caught while feeding on animals, whereas others were manually caught in the environment.

**Table 3:** Location and capture method used for the nonsystematic horse fly collections.

Identifier	Location	Department	Capture method
Manually 7	31°42″29.0″S, 55°48′09.1″W	Tacuarembó	Manually (from the environment)
Manually 8	31°21′37.8″S, 56°05′14.6″W	Tacuarembó	Manually (feeding on cattle)
Manually 9	31°39′59.8″S, 55°57′32.5″W	Tacuarembó	Manually (from the environment)
Manually 10	31°28′29.4″S, 57°53′44.4″W	Paysandú	Manually (feeding on cattle)
NZI 11	31°28′29.4″S, 57°53′44.4″W	Paysandú	NZI trap
NZI 12	34°17′30.2″S, 57°37′41.4″W	Colonia	NZI trap
Manually 13	34°18′14.1″S, 57°31′42.7″W	Colonia	Manually (feeding on cattle)
NZI 14	34°18′14.1″S, 57°31′42.7″W	Colonia	NZI trap

### Statistical analysis

To perform descriptive and inferential analyses, two databases were created in Excel: one consisted of the systematic captures, and the other was composed of the nonsystematic captures. After the identification of any data errors, these databases were then imported into STATA 2014^28^.

The datasets generated during and/or analyzed during the current study are available^29^.

#### Descriptive analysis

Descriptive analyses were performed based on the total number of individuals captured by location, date, season, trap type, type of manual capture (environmental or on animals) and species.

#### Inferential analysis

The influence of the mt and environment (lowland and highland) on the abundance of horse fly species was tested using generalized linear models (GLMs). The models were obtained by the extraction of significant terms (p < 0.05) from the full model, which included all of the variables, namely, mt, maximum temperature, minimum temperature, relative humidity, rainfall, and environment, and their interactions^30^. Each term was analyzed by ANOVA and chi-square tests (Chi) to recalculate the deviation explained by the other terms.

We tested the hypothesis that the mt in different environments alters the abundance of Tabanidae using models with response variables that assume integer values with regard to the abundance. The explanatory variables used in this analysis were the mt and environment without any interaction terms. The complete models that were used for hypothesis testing were as follows:

$${\rm{Abundance}}={\rm{mt}}+{\rm{environment}}$$

The different years in which the experiments were conducted were not used as explanatory variables due to the autocorrelation between the mt and year. In the models, a plus sign (+) denotes the addition of a variable. A quasi-Poisson error distribution and the log-link function were used to model the estimated abundance.

## Supplementary information

Supplementary Table 1.

## References

[1] Baldacchino F (2014). Tabanids: Neglected subjects of research, but vectors of disease agents Important!. Infect. Genet. Evol..

[2] Foil LD, Hogsette JA (1994). Biology and Control of Tabanids, stable flies and horn flies. Rev. Sci. Tech..

[3] Perich MJ, Wright RE, Lusby KS (1986). Impact of horse flies (Diptera: Tabanidae) on beef-cattle. J. Econ. Éntomol.

[4] Krinsky WL (1976). Animal-disease agents transmitted by horse flies and deer flies (Diptera, Tabanidae). J. Med. Éntomol.

[5] Foil LD (1989). Tabanids as vectors of disease agents. Parasitol. Today.

[6] Magnarelli LA, Anderson JF (1980). Feeding-behavior of Tabanidae (Diptera) on cattle and serological analysis of partial blood meals. Env. Éntomol.

[7] Scoles G, Miller A, Foil L (2008). Comparison of the Efficiency of Biological Transmission of *Anaplasma marginale* (Rickettsial: Anaplasmataceae) by *Dermacentor andersoni* Stiles (Acari: Ixodidae) With Mechanical Transmission by the Horse Fly, *Tabanus fuscicostatus* Hine (Diptera: Muscidae). J. Méd. Éntomol.

[8] INIA. GRAS INIA data, http://www.inia.uy/gras/Clima/Banco-datos-agroclimatico (2019).

[9] Chvála, M., Lyneborg, L. & Moucha, J. The Horse Flies of Europe (Diptera, Tabanidae). Entomological Society of Copenhagen, Copenhagen ISBN: 978-09-00-84857-5 (1972).

[10] Krüger Rodrigo Ferreira, Krolow Tiago Kütter (2015). Seasonal patterns of horse fly richness and abundance in the Pampa biome of southern Brazil. Journal of Vector Ecology.

[11] Hornok S (2008). Molecular identification of *Anaplasma marginale* and rickettsial endosymbionts in blood-sucking flies (Diptera: Tabanidae, Muscidae) and hard ticks (Acari: Ixodidae). Vet. Parasitol..

[12] Thorsteinson AJ, Bracken GK, Hanec W (1965). The orientation of horse flies and deer flies (Tabanidae, Diptera). III. The use of traps in the study of orientation of Tabanids in the field. Éntomol Exp. Appl..

[13] Thompson Patrick H. (1969). Collecting Methods for Tabanidae (Diptera)1. Annals of the Entomological Society of America.

[14] Mihok S. (2002). The development of a multipurpose trap (the Nzi) for tsetse and other biting flies. Bulletin of Entomological Research.

[15] VAN HENNEKELER K., JONES R. E., SKERRATT L. F., FITZPATRICK L. A., REID S. A., BELLIS G. A. (2008). A comparison of trapping methods for Tabanidae (Diptera) in North Queensland, Australia. Medical and Veterinary Entomology.

[16] Coscarón S, Papavero N (2009). Catalogue of Neotropical Diptera. Tabanidae. Neotropical Diptera.

[17] Coscarón S, Martinez M (2019). Checklist of tabanidae (Insecta: Diptera) from Uruguay. J. Éntomol Soc. Argentina.

[18] Hawkins JA, Love JN, Hidalgo RJ (1982). Mechanical transmission of Anaplasmosis by tabanids (Diptera, Tabanidae). Am. J. Vet. Res..

[19] Henriques AL, Krolow TK, Rafael JA (2012). Corrections and additions to Catalog of Neotropical Diptera (Tabanidae) of Coscarón & Papavero (2009). Rev. Brasileira de. Entomologia.

[20] Coscarón, S. Tabanidae (Diptera-Insecta). In: Morrone, J. J. & Coscarón, S. (ed.) Biodiversidad de artrópodos argentinos: una perspectiva biotaxonómica Sur, La Plata, pp 341–352 (1998).

[21] Catálogo Taxonômico da Fauna do Brasil, http://fauna.jbrj.gov.br/fauna/listaBrasil/ConsultaPublicaUC/ConsultaPublicaUC.do Accessed 04 August 2019 (2019).

[22] Burnett AM, Hays KL (1974). Some influences of meteorological factors on flight activity of female horse flies (Diptera: Tabanidae). Env. Éntomol.

[23] Gorayeb IS (1995). Tabanidae (Diptera) da Amazônia. XI. Sazonalidade espécies das da Amazônia Oriental e correlação com fatores climáticos. Bol. Mus. Para. Emílio Goeldi.

[24] Cardenas RE, Hernandez NL, Barragán AR, Dangles O (2012). Differences in morphometry and activity Among tabanid fly assemblages in an Andean tropical montane cloud forest: indication of altitudinal migration. Biotropica.

[25] Barros ATM (2001). Seasonality and relative abundance of Tabanidae (Diptera) captured on horses in the Pantanal, Brazil. Mem. Inst. Oswaldo Cruz.

[26] Gorayeb, I. S. Tabanidae (Diptera) da Amazônia. XVI – Atividade diurna de hematofagia de espécies da Amazônia Oriental, em áreas de mata e pastagem, correlacionada com fatores climáticos. *Bol Mus Paraense Emílio Goeldi* **16**, 23–63, http://repositorio.museu-goeldi.br:8080/jspui/handle/mgoeldi/1021 (2000).

[27] Vale GA, Lovemore DF, Flint S, Cockbill GG (1988). Odour-baited targets to control tsetse flies, *Glossina* spp. (Diptera: Glossinidae), in Zimbabwe. Bull. Éntomol Res..

[28] StataCorp. Stata Statistical Software: Release 14. College Station, T.X.: StataCorp L.P (2015).

[29] Miraballes, C. *et al*. Data for “Diversity and seasonality of horse flies (Diptera: Tabanidae) in Uruguay”, Mendeley Data, v2, 10.17632/zstbdddgv8.2 (2019). 10.1038/s41598-019-57356-0 PMC6962385 31942013

[30] Crawley, M. J. The R Book, John Wiley, New York, 942 p (2007).

