# Supplementary material for: Diversity and seasonality of horse flies (Diptera: Tabanidae) in Uruguay
Source: Sci Rep. 2020 Jan 15;10:401. doi: 10.1038/s41598-019-57356-0 (PMC6962385; doi:10.1038/s41598-019-57356-0)
Supplement: Supplementary file 1 — Supplementary Table 1. [file 41598_2019_57356_MOESM1_ESM.docx]

**Diversity and seasonality of horse flies (Diptera: Tabanidae) in Uruguay**

Martín Lucas^1,2^; Tiago K. Krolow^3^; Franklin Riet-Correa^1^; Antonio Thadeu M. Barros^4^; Rodrigo F. Krüger^5^; Anderson Saravia^1^; Cecilia Miraballes^1*^

^1^Instituto Nacional de Investigación Agropecuaria (INIA), Plataforma de Salud Animal, Tacuarembó, Uruguay, Casilla de Correo 78086, CP 45000 Uruguay.

^2^Facultad de Veterinaria, Universidad de la República (UDELAR), Alberto Lasplaces 1620, CP 11600, Montevideo, Uruguay

^3^Universidade Federal do Tocantins – UFT, Rua 03, Qd 17, S/N, Bairro Jardim dos Ipês, Porto Nacional, TO, Brazil

^4^ Embrapa Beef Cattle, Campo Grande, MS, Brazil

^5^Universidade Federal de Pelotas, Instituto de Biologia, Departamento de Microbiologia e Parasitologia, Campus Universitário Capão do Leão, s/nº, Pelotas, RS, Brazil

*email [cmiraballes@inia.org.uy](mailto:cmiraballes@inia.org.uy)

| **Supplementary Table 1. Climatic variables that were recorded and evaluated throughout the study period** | | | | | |
| --- | --- | --- | --- | --- | --- |
| **Year** | **Month** | **Average maximum temperature (ºC)** | **Average minimum temperature (°C)** | **Accumulated rainfall (mm)** | **Average mean relative humidity (%)** |
| **2017** | Sep | 20.5 | 12.6 | 210.4 | 85 |
| **2017** | Oct | 21.9 | 12.1 | 214.8 | 75 |
| **2017** | Nov | 25.5 | 12.2 | 45.1 | 64 |
| **2017** | Dec | 30.6 | 16.2 | 38.6 | 58 |
| **2018** | Jan | 31.4 | 18.2 | 37.3 | 56 |
| **2018** | Feb | 30.3 | 16.6 | 64.9 | 61 |
| **2018** | Mar | 27.4 | 13.7 | 64.0 | 65 |
| **2018** | Apr | 27.0 | 16.2 | 62.1 | 77 |
| **2018** | May | 19.5 | 11.1 | 207.6 | 83 |
| **2018** | Jun | 15.2 | 6.0 | 29.3 | 83 |
| **2018** | Jul | 15.2 | 8.0 | 193.0 | 85 |
| **2018** | Ago | 16.2 | 6.8 | 147.3 | 82 |
| **2018** | Sep | 21.3 | 12.7 | 250.7 | 81 |
| **2018** | Oct | 22.3 | 11.7 | 35.0 | 73 |
| **2018** | Nov | 26.2 | 14.8 | 99.1 | 71 |
| **2018** | Dec | 27.2 | 15.4 | 177.2 | 73 |
| **2019** | Jan | 28.0 | 19.4 | 362.7 | 80 |
| **2019** | Feb | 27.9 | 18.6 | 111.8 | 76 |
| **2019** | Mar | 26.0 | 16.2 | 40.3 | 78 |
| **2019** | Apr | 24.1 | 15.1 | 133.7 | 81 |
| **2019** | May | 19.0 | 11.8 | 99.1 | 90 |
| **2019** | Jun | 20.4 | 10.5 | 91.3 | 86 |
